# The STAT5b Pathway Defect and Autoimmunity

**DOI:** 10.3389/fimmu.2012.00234

**Published:** 2012-08-14

**Authors:** Takahiro Kanai, Jennifer Jenks, Kari Christine Nadeau

**Affiliations:** ^1^Division of Immunology and Allergy, Department of Pediatrics, School of Medicine, Stanford UniversityStanford, CA, USA

**Keywords:** allergy, autoimmunity, IL-2, immunodeficiency, STAT5b, CD25, Foxp3, Bcl-2

## Abstract

The signal transducer and activator of transcription (STAT) 5b is a universal transcription factor that plays key biological roles in allergic diseases, immunodeficiencies, autoimmunities, cancers, hematological diseases, growth disorders, and lung diseases. The identification of distinct pathological manifestations of STAT5b deficiency in humans has highlighted the critical role of the STAT5b pathway. Proper gene transcription at *IL-2R* α, *FOXP3*, *Bcl-2*, and growth hormone (GH) associated loci are thought to be associated with normal STAT5b transcriptional activity. These genes are thought to play important roles in allergy/autoimmunity, immunodeficiency, cancer/anemia, and growth, respectively. The *STAT5A* and *STAT5B* genes are collocated on 17q11. Although these two monomeric proteins exhibit peptide sequence similarities of >90%, it is known through observations of STAT5b deficient subjects that STAT5a and STAT5b are not fully redundant in humans. Patients with STAT5b deficiency have decreased numbers of regulatory CD4^+^CD25^high^ T cell (Treg) despite their STAT5a levels being normal. Prior studies on STAT5b deficient subjects have revealed immunological aberrations associated with the following disease phenotype: modest lymphopenia and decreased populations of Treg, γ−δ T cells, and natural killer (NK) cells. Most subjects with STAT5b deficiency show severe eczema, and autoimmune disease (juvenile idiopathic arthritis, autoimmune thyroiditis, idiopathic thrombocytic purpura) which are thought to be associated with Treg dysfunction. We will review the likely pathophysiological mechanisms associated with STAT5b deficiency.

## Introduction

The signal transducer and activator of transcription (STAT) 5b is a universal transcription factor that plays key biological roles in allergic disease, immunodeficiencies, autoimmunities, cancers, hematological disease, growth disorders, and lung disease (Buggins and Pepper, 2010; Nadeau et al., 2011).

There are several differences between human and mouse in the roles of STAT5b (Nadeau et al., 2011). The identification of STAT5b deficiency in humans, and the distinct and destructive pathology associated with this deficiency has highlighted the critical role the STAT5b pathway. Research on the immunologic function of STAT5b has demonstrated its importance for the *in vivo* accumulation of regulatory CD4^+^CD25^high^ T cells (Treg) with immunoregulatory function (Cohen et al., 2006; Nadeau et al., 2011). The specific role that STAT5b plays in the pathogenesis of the aforementioned diseases has led to suggestions that the transcription factor might have potential as a novel diagnostic and/or therapeutic target in some disease settings.

In this review, we summarize recent advances in our understanding of the STAT5b pathway in human mainly as well as the autoimmune manifestations induced by the defects within it.

## The STAT5b Pathway

### STAT5b gene and protein, and non-redundancy between STAT5a and STAT5b

**Figure 1:**
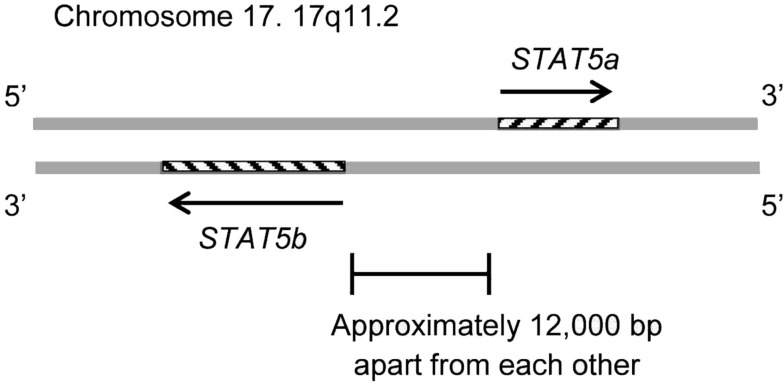
**The STAT5B gene is collocated on 17q11.2 approximately 12 kb apart from the STAT5A gene**. The STAT5B gene is on the negative strand, and the STAT5a gene is on the positive strand. The genomic size of STAT5b is 58,700–77,229. The genomic size of STAT5a is approximately 24,000.

The *STAT5B* gene is collocated on 17q11.2 approximately 12 kb apart from *STAT5A* (Figure 1). Both genes are regulated by a Sp-1 cis-element (Crispi et al., 2004).

Although STAT5a and STAT5b show peptide sequence similarities of >90%, they differ by six amino acid in the DNA binding domain and 20 amino acids in their carboxy termini (Boucheron et al., 1998; Grimley et al., 1999; Soldaini et al., 2000; Wei et al., 2008). Additional reports of a common disease phenotype specifically associated with STAT5b deficiency in humans (but no such phenotype associated with STAT5a deficiency) indicates that, at least in humans, the roles of STAT5a and STAT5b are not fully redundant (Nadeau et al., 2011).

Structural dissimilarities between the STAT5a and the STAT5b on transactivation domains or subtle differences in the DNA binding affinities of STAT5 dimer pairs could influence gene regulation, but cell-dependent asymmetries in the availability of phosphorylated STAT5a or STAT5b could also another factor. Signal attenuation by phosphatase action or classic feedback inhibition, or truncated forms of STAT5b lacking in transactivation capacity, may compete upstream for activation and diminish access of full length molecules to DNA binding sites (Grimley et al., 1999). Thus, both STAT5 proteins could bind to the same targets, and any differences between STAT5a and STAT5b may arise from differential expression or difference in kinetics of DNA binding (Grimley et al., 1999).

### Upstream of STAT5b: Cytokines and their receptors

**Figure 2:**
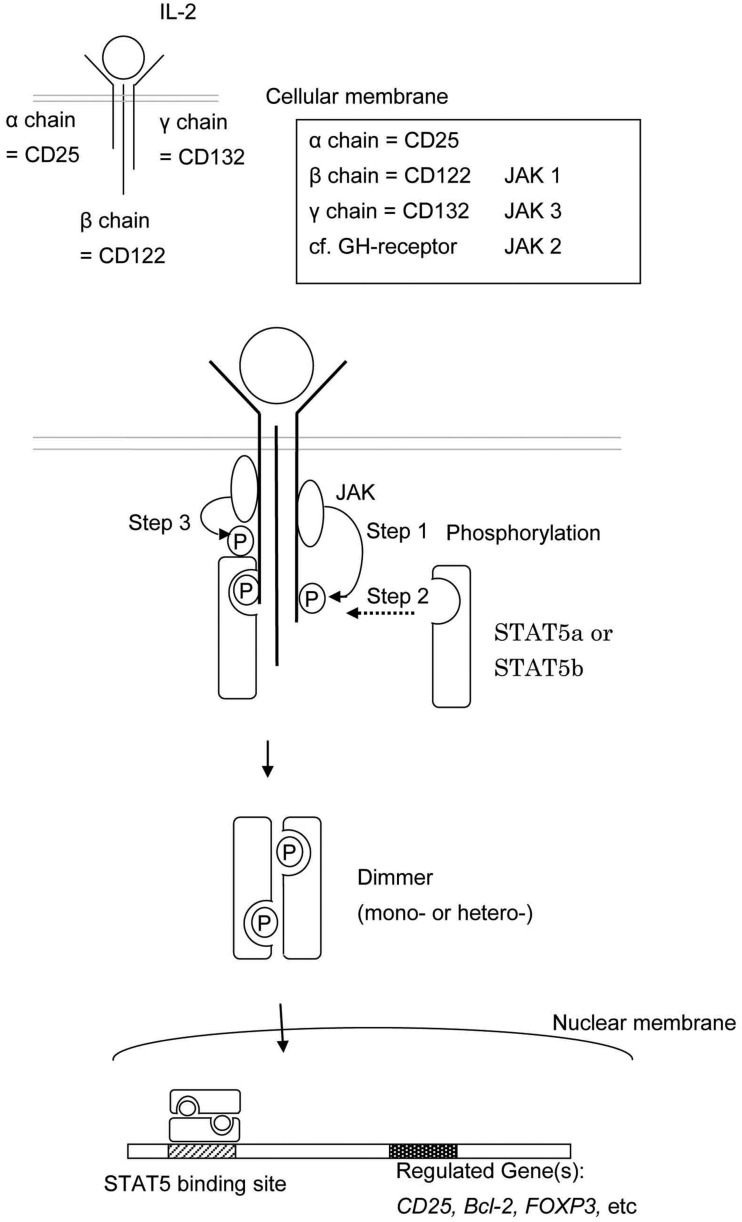
**This shows the schema of STAT5a and/or STAT5b activation**. The engagement between a cytokine and its cell surface receptor results in subsequent activation of receptor-associated JAK. Activated JAK phosphorylates specific tyrosine resides in the cytoplasmic domain of the receptor which in turn serves as the docking sites for STAT5a and/or STAT5b. STAT5a and/or STAT5b are recruited to the phosphorylated receptor and subsequently phosphorylated by JAKs. The phosphorylated STAT5a and/or STAT5b dimerize, leave the receptor, and translocate to the nucleus.

Signal transducer and activator of transcription 5b is a common downstream effector of the IL-2, -4, -7, -9, -13, -15, -21, growth hormone (GH; Liu et al., 1997), erythropoietin, thrombopoietin, and granulocyte colony-stimulating factor signaling molecules (Nadeau et al., 2011). Each cytokine has associated receptors, and each receptor has associated Janus kinases (JAK). For example, the IL-2 receptor is composed of an α chain (CD25), β chain (CD122), and γ chain (CD132; Lin and Leonard, 2000). The β chain is associated with JAK1 and JAK3 (Zhu et al., 1998) and the γ chain is associated with JAK3 (Figure 2; Russell et al., 1995). The growth hormone receptor (GHR) is associated with JAK2 (Hwa et al., 2011).

The CD25 plays an important role as an integral component of the high affinity IL-2 receptor. Its ligand, IL-2, is a cytokine known for the role it plays in lymphocytic function, especially with relation to T cell biology. There are two functional receptors for IL-2: one is a heterodimeric complex formed by the β and γ chains, while the other is a trimeric membrane-spanning complex composed of the α, β, and γ subunits. The latter receptor has a higher affinity for IL-2 than the former (Lin and Leonard, 2000). Additionally, defects in STAT5b expression and function have been shown to result in reduced expression of IL-2Rα, thereby potentially limiting cellular response to IL-2 signaling (Cohen et al., 2006).

The engagement between cytokines and their cell surface receptors results in subsequent activation of receptor-associated JAK tyrosine kinase activity. Activated JAKs phosphorylate specific tyrosine resides in the cytoplasmic domain of their associated receptor, and these newly phosphorylated residues serve as docking sites for STAT proteins (Figure 2; Grimley et al., 1999).

### Phosphorylation of STAT5b by JAKs (mainly JAK1 and 3)

Intracellular signal transduction pathways are essential for transforming extracellular cytokine signaling into appropriate cellular responses. The phosphorylation of STAT molecules is a key component in the JAK/STAT signal transduction pathway (Xu and Qu, 2008).

**Figure 3:**
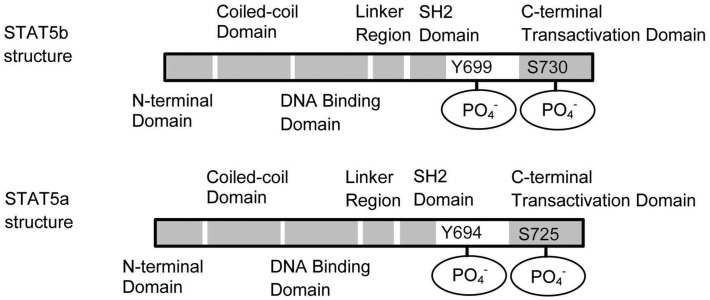
**Schematic structures of STAT5a and STAT5b**. STAT5a and STAT5b differ in the C terminal domain. The dimerization occurs through the interaction between the SH2 domains.

Cytokine engagement of membrane-associated receptors brings receptor subunits into proximal relationships necessary for JAK autophosphorylation (Figure 2). Cytoplasmic STAT monomers are subsequently able to bind the phosphotyrosine residues on engaged cytokine receptors through the highly conserved SH2 domain located on all proteins of the STAT family (Figure 3).

As a result of this docking, JAK and STAT molecules are brought into close enough proximity to allow for JAK phosphorylation, and therefore activation, of STAT molecules. In the case of STAT5, phosphorylated STAT5a and/or STAT5b then homo- or hetero-dimerize (sometime tetramaerization; John et al., 1999; Soldaini et al., 2000; Mandal et al., 2011) by each SH2 domain, leave the receptor, and translocate to the nucleus where they act as a transcriptional activator for each target gene (Levy and Darnell, 2002).

### Downstream of the STAT5b pathway

Signal transducer and activator of transcription 5b dimers translocate into the nucleus and bind to specific regions thought to be associated with transcription of *FOXP3*, *CD25*, *Bcl-2*, *IGF-1* (Nadeau et al., 2011). Reports indicate that STAT5b may preferentially interact with different DNA binding sites depending on the cell type considered.

#### Fork-head box P3 (FOXP3): a key transcription factor essential for Treg cell development and function

The transcription factor FOXP3 is critical for the thymic development of Tregs (Sakaguchi et al., 2008). In mice, CD4^+^CD25^+^ peripheral T cells and CD4^+^CD25^+^CD8^−^ thymocytes express Foxp3 and are considered to be immunoregulatory, whereas other thymocytes/T cells, either in a resting or activated state, do not (Fontenot et al., 2003; Hori et al., 2003; Khattri et al., 2003; Sakaguchi et al., 2008).

Studies investigating the effects of FOXP3 suppression report complications associated with Treg dysfunction to be a main pathological consequence. Mutations of the *FOXP3* gene were found to be the cause of an IPEX (immune dysregulation, polyendocrinopathy, enteropathy, X-linked syndrome), which is characterized by autoimmune disease in multiple endocrine organs (as in type I diabetes and thyroiditis), inflammatory bowel disease, and severe allergy (Chatila et al., 2000; Bennett et al., 2001; Wildin et al., 2001). Deletion or dysfunction of FOXP3 causes impaired function and/or homeostasis of Tregs, and has been implicated in the development of several common autoimmune and inflammatory diseases (Campbell and Koch, 2011).

#### The essential role of CD25 in Treg development and function

High expression of CD25 is considered to be a marker of Tregs (Sakaguchi et al., 1995) and studies have elaborated on this concept, demonstrating that the IL-2Rα serves not only as a marker for natural Treg, but also, as a protein essential for its development and function (Sakaguchi et al., 2008). The importance of CD25 in the development of a normal immune response is emphasized by the finding that a truncation mutant of CD25 results in an immunodeficiency in humans characterized by an increased susceptibility to viral, bacterial, and fungal infection (Sharfe et al., 1997). In addition, gene targeting analysis also reveals that CD25 deficient mice exhibit autoimmunity (Willerford et al., 1995).

While CD25 contributes to IL-2 binding affinity and not to the recruitment of signaling molecules (Lin and Leonard, 2000) its role as a component of the high affinity IL-2 receptor makes it indispensable for the activation of cell signaling pathways associated with IL-2 signal transduction (Sakaguchi et al., 2008).

#### Bcl-2 is an apoptotic inhibitor protein

Bcl-2 is an apoptosis inhibitor protein. Most cell death in vertebrates occurs via the mitochondrial pathway of apoptosis, in which Bcl-2 and other anti-apoptotic proteins (Bcl-xL, Bcl-w, Mcl-1, and Bfl-1/A1) are key effectors (Llambi and Green, 2011). Aberrant regulation of Bcl-2 has been reported to cause or correlate with autoimmunity or cancer, particularly leukemia (Buggins and Pepper, 2010; Tischner et al., 2010). Deletion of self-reactive immune cells occurs through this apoptotic pathway and is necessary for the maintenance of immune tolerance (Tischner et al., 2010). Overexpression of Bcl-2 has been noted in patients with systemic lupus erythematosus (Tischner et al., 2010). In malignant diseases, decreased rate of apoptotic cell death is also found to be responsible in the proliferative process (Ulukaya et al., 2011).

#### Insulin-like growth factor-I and Insulin-like growth factor binding protein-3 play an important role in fat metabolism and skeletal development

Insulin-like growth factor-I promotes skeletal development and fat metabolism, and insulin-like growth factor binding protein-3 (IGFBP-3) acts as a negative regulator for IGF-I signaling (Kawai and Rosen, 2010).

The activation of IGF-I is initiated by the interaction of circulating GH with the GHR. The cytoplasmic domain of GHR associates preferentially with JAK2. Activation of JAK2 by GHR engagement leads to the activation of STAT5b (Hwa et al., 2011).

In humans, serum IGF-I concentrations have a positive correlation with skeletal mass (Langlois et al., 1998). A report on the disease characteristics of STAT5b deficiency in humans highlights low serum IGF-1 as one defining clinical feature of the disease (Hwa et al., 2004; Nadeau et al., 2011). STAT5b deficient patients also exhibit stunted growth and poor response to GH therapy (Nadeau et al., 2011).

IGF-I was also reported as a critical factor for adipogenesis (Kawai and Rosen, 2010). The lack of this factor results in a defect in adipose tissue formation by mitogen-activated protein kinase deactivation in conjunction with GH (Boney et al., 2000; Hwa et al., 2011).

IGFBP-3 suppresses adipogenesis independent of IGF-I binding (Chan et al., 2009) and reduces bone mineral density (Kawai and Rosen, 2010).

### Human STAT5b pathway defect and autoimmunity

Human STAT5b deficiency is a recently identified, rare autosomal recessive disease that involves both severe GH-resistant growth failure and severe primary immunodeficiency. It was first discovered in patients with dwarfism associated with normal levels of serum GH, but very low levels of IGF-I (Kofoed et al., 2003; Bernasconi et al., 2006; Chia et al., 2006). Affected individuals also exhibited recurrent infections, chronic diarrhea, eczema, and/or lymphocytic interstitial pneumonitis (Kofoed et al., 2003; Bernasconi et al., 2006; Chia et al., 2006). Immunophenotyping of these patients have revealed modest lymphopenia and decreased populations of Treg, γ−δ T cells, and natural killer (NK) cells (Bernasconi et al., 2006; Cohen et al., 2006). There are currently 10 published cases of STAT5b deficiency (Table 1; Nadeau et al., 2011). Ongoing research efforts aim to identify the molecular mechanisms of STAT5b in postnatal growth and immunity.

**Table 1:** **Demographics of published cases with STAT5b deficiency**.

	#1	#2	#3	#4	#5	#6	#7	#8	#9	#10	Reference values
STAT5b Mutation	*A630P*	*1191insG*	*R152X*	*R152X*	*1103insC*	*1680delG*	*1680delG*	*424_427del*	*424_427del*	*F646S*	
	*−/−*	*−/−*	*−/−*	*−/−*	*−/−*	*−/−*	*−/−*	*−/−*	*−/−*	*−/−*	
Place of origin	Argentina	Turkey	Argentina	Argentina	Caribbean	Kuwait	Kuwait	Brazil	Brazil	Argentina	
Sex	F	F	F	F	M	F	F	M	M	F	
Height, SDS	*−*7.5	*−*7.8	*−*9.9	*−*5.3	*−*5.9	*−*5.8	*−*5.6	*−*5.6	*−*3	-5.90	
Age	16.5	16.4	15.3	12	31	2	4	6	2	18	
Onset of chronic pulmonary disease	7 years	7 years	1 year	6 months	No	6 years	6 years	Yes	Yes	No	
Skin pathology	Eczema	Eczema	Eczema	Eczema	Ichytosis	No	No	Atopic	Atopic	Seborrheic dermatitis	
Autoimmune manifestations	+Abs to BEC	ITP + Abs to platelets	No	AIT with + Abs to thymoglobulin	Ichytosis + Abs to platelets	SJIA	No	No	No	AIT	
Parental consanguinity	Yes	Yes	NA	Adopted	NA	Yes	Yes	ND	ND	Adopted	
Paternal height, SDS	*−*0.3	*−*0.9	*−*2.2	NA	*−*0.8	*−*1.28	*−*1.28	*−*1.6	*−*1.6	NA	
Mother height, SDS	*−*1.2	*−*0.6	*−*3.3	NA	*−*2.8	*−*0.6	*−*0.6	*−*1.3	*−*1.3	NA	
Birth weight/length (cm)	1400/ND	2350/49	2500/ND	1650/ND	3270/50	2400/ND	3600/ND	1650/39	2400/49	2250/44	
Puberty	Delayed	Delayed	Delayed	NA	Delayed	NA	NA	NA	NA	NA	
**BIOCHEMISTRY**											
GH basal (ng/mL)	9.4	14.2	6.6	1.8	0.13	17.7	5.7	1.7	1	1.7	
GH stimulated (ng/mL)	53.8	NA	NA	12.5	14.2	NA	NA	20.6	14	27.1	>6.0
IGF-I stimulated (ng/mL)	38	7.0	<10	0.8	14	<5	<5	34	<25	16	119–483
IGFBP-3 (ng/mL)	874	543	NA	500	180	700	800	520	750	840	210-740
ALS (acid labile subunit; mg/L)	2.9	1.2	NA	0.7	0.7	0.4	0.8	520	750	NA	
References for published case	Cohen et al. (2006), Kofoed et al. (2003)	Hwa et al. (2005)	Bernasconi et al. (2006)	Bernasconi et al. (2006)	Vidarsdottir et al. (2006)	Hwa et al. (2007)	Hwa et al. (2007)	Pugliese-Pires et al. (2010)	Pugliese-Pires et al. (2010)	Scaglia et al. (2012)	

*ITP, idiopathic thrombocytopenic purpura; AIT, autoimmune thyroiditis; Abs, antibodies; BEC, bronchial epithelial cells; SJIA, systemic juvenile idiopathic arthritis; ND, not diagnosed; NA, not currently available; PC, personal communication. The data of #6 and #7 in adjacent columns represent data from siblings*.

*Heterozygote STAT5b-deficient subjects who are relatives of homozygote STAT5b-deficient subjects currently have not been identified to have any abnormal clinical phenotypes, although a specific detailed review of their clinical demographics and laboratory studies has not been done yet*.

#### Previous cases

The first case of a STAT5b mutation was reported in 2003, in a 16-year-old female with severe growth retardation (−7.5 SD) and pulmonary complications (Kofoed et al., 2003). The reported missense mutation (p.A630P) disrupted the core of anti-parallel β-sheets that enable phosphate-binding, causing aberrant folding (Chen et al., 1998) aggregation of mutant STAT5b protein, and loss of thermodynamic stability (Chia et al., 2006; Fang et al., 2006). The patient presented with early onset lymphocytic interstitial pneumonitis, chronic lung disease, hemorrhagic varicella, atopy, and autoimmune disease (Kofoed et al., 2003). At age 7, she developed lymphocytic interstitial pneumonia and after receiving potent immunosuppressive therapy, had two major infectious complications – severe varicella-zoster virus infection and *Pneumocystis jiroveci* pneumonia. Another biopsy at age 10 also indicated lymphoid interstitial pneumonia, and *P. carinii* was isolated from the tissue. Later studies revealed decreased numbers of Treg and reduced Treg suppressive function (Cohen et al., 2006).

In 2005, a second case of a STAT5b deficiency was identified in a 16-year-old Turkish female with severe growth failure, GHI, atopic dermatitis, pruritic skin lesions, primary idiopathic pulmonary fibrosis with diffuse lung involvement, and autoimmune disease, as well as bleeding diathesis caused by defective thrombocyte aggregation, preventing a potential lung biopsy (Hwa et al., 2005). Sequencing of the *STAT5b* gene revealed a novel homozygous frameshift mutation (c.1191insG) that led to protein termination (p.N398EfsX16) and consequent lack of immunodetectable STAT5b protein (Hwa et al., 2005).

Another case was identified in 2006 in a 16-year-old female with severe postnatal growth failure, GHI, and immunodeficiency (Bernasconi et al., 2006). Pulmonary-function tests showed mixed, restrictive, and obstructive moderate ventilative insufficiency, but no lung biopsy was performed. Notably, this case was the first to identify a role for STAT5b not only in the human GH signaling cascade, but also in the cytokine-mediated immune response. The STAT5b deficient patient had moderate T cell lymphopenia, normal CD4/CD8 ratios, and very low numbers of NK cells and γ−δ T cells, and the T cells presented a chronically hyperactivated phenotype (Bernasconi et al., 2006).

Since 2012, five other mutations have been published on a total of seven additional subjects (Table 1). Lung pathology has been common among these patients (8 of 10), but of these remaining seven subjects, only a few have received lung biopsies. A STAT5b deficient male with the mutation 424_427del received a biopsy at 6 years of age that indicated severe lymphocytic interstitial pneumonitis. Considered together, these studies have firmly established a correlation between STAT5b deficiency and immune dysfunction, in addition to GHI and severe growth problems.

#### Clinical manifestations and diagnosis

Signal transducer and activator of transcription 5b deficiency should be considered in the differential diagnosis of a patient who has normal gestational growth and birth size but acquires significantly short stature and recurrent infections. This pattern of growth is typical of patients with GHI. Height may range from −3.0 to −9.9 SD in girls and boys, respectively (Table 1).

Regarding hormone evaluations, all described patients have had normal levels of GH at baseline, but after stimulation, GH concentrations were often elevated (Table 1). In contrast, serum IGF-I, IGFBP-3, and acid labile subunit concentrations were low, and even upon administration of GH, remained low. Elevated prolactin levels were also observed in patients with recorded concentrations.

Most patients have displayed evidence of immune dysfunction, including atopic disease, chronic lung disease, viral infections, and/or autoimmune diatheses. Often present in childhood, severe pulmonary disease is of particular concern, as it has affected 8 of the 10 known STAT5b deficient patients and two patients have died of respiratory failure. For all cases of lung pathology except for that of Patient #5, an axial chest CT scan has shown increased interstitial patterns and ground-glass appearance. These pulmonary lesions are T cell predominant, despite peripheral lymphopenia. In most cases, severe eczema, thrombocytopenic purpura, and/or autoimmune disease, such as juvenile idiopathic arthritis, were present in addition to severe lung disease. However, it should be noted that 1 of the 10 subjects to date has less severe immune dysfunction. Congenital ichthyosis was diagnosed at birth, and the patient had hemorrhagic varicella at 16 years of age but had no history of pulmonary of immunological problems (Vidarsdottir et al., 2006).

Previous immunological studies have established the importance of STAT5b proteins in the development, homeostasis, and proliferation of different lymphocyte populations. Immune repertoires of STAT5b deficient patients have shown moderate lymphopenia, with very low numbers of NK and T cells, as well as Treg dysfunction. Furthermore, B cell populations and immunoglobulin G levels in at least patient are normal to elevated, as consistent with autoimmune disease symptoms (Cohen et al., 2006).

#### Disease management

In order to improve clinical outcomes for patients with STAT5b deficiency, optimizing early diagnosis in these patients is critical. To date, overall management of STAT5b deficiency is still unclear. GH therapy is ineffective due to the patients’ GHI. It is presumed that IGF-I therapy may be an effective treatment, unless the presence of chronic infection limits the growth response. However, to date, no clinical trials of IGF-I therapy have been performed in these patients.

Patients should be closely monitored for signs and symptoms of immunodeficiency. Infections such as severe varicella or recurrent pneumonias should be aggressively treated with appropriate antimicrobial therapies. Patients with autoimmune conditions, atopic diseases, or pulmonary fibrosis may also require antiproliferatives or immunosuppressants, such as steroids, to address overactive effector T cell responses. Because severe chronic lung disease in this patient population often leads to high morbidity and mortality, patients should be carefully monitored with pulmonary-function tests and physical examinations, which may improve treatment options to decrease the lung disease severity.

Although current management of STAT5b deficiency is primarily dictated by specific end-organ pathology, current research is addressing the possibility of enhancing STAT5b and/or STAT5a pathways (Zeiser et al., 2008; Strauss et al., 2009). Future therapy may be expected to prevent and reflect rationally based drug design to enhance certain drug targets in the STAT5b and/or STAT5a pathways.

## Conclusion

In this review, we focused on the STAT5b pathway and the mechanisms by which defects in protein structure and or expression might result in autoimmunity. A better understanding of STAT5b and its distinct biological functions is necessary for the development of new diagnostic and therapeutic approaches for treating patients suffering from its deficiency.

## Conflict of Interest Statement

The authors declare that the research was conducted in the absence of any commercial or financial relationships that could be construed as a potential conflict of interest.

## References

[B1] Bennett C. L. Christie J. Ramsdell F. Brunkow M. E. Ferguson P. J. Whitesell L. Kelly T. E. Saulsbury F. T. Chance P. F. Ochs H. D. (2001). The immune dysregulation, polyendocrinopathy, enteropathy, X-linked syndrome (IPEX) is caused by mutations of FOXP3. Nat. Genet. 27, 20–21 10.1038/83713 11137993

[B2] Bernasconi A. Marino R. Ribas A. Rossi J. Ciaccio M. Oleastro M. Ornani A. Paz R. Rivarola M. A. Zelazko M. Belgorosky A. (2006). Characterization of immunodeficiency in a patient with growth hormone insensitivity secondary to a novel STAT5b gene mutation. Pediatrics 118, e1584–e1592 10.1542/peds.2005-2882 17030597

[B3] Boney C. M. Gruppuso P. A. Faris R. A. Frackelton A. R. Jr. (2000). The critical role of Shc in insulin-like growth factor-I-mediated mitogenesis and differentiation in 3T3-L1 preadipocytes. Mol. Endocrinol. 14, 805–813 10.1210/me.14.6.805 10847583

[B4] Boucheron C. Dumon S. Santos S. C. Moriggl R. Hennighausen L. Gisselbrecht S. Gouilleux F. (1998). A single amino acid in the DNA binding regions of STAT5A and STAT5B confers distinct DNA binding specificities. J. Biol. Chem. 273, 33936–33941 10.1074/jbc.273.51.33936 9852045

[B5] Buggins A. G. Pepper C. J. (2010). The role of Bcl-2 family proteins in chronic lymphocytic leukaemia. Leuk. Res. 34, 837–842 10.1016/j.leukres.2010.03.011 20359747

[B6] Campbell D. J. Koch M. A. (2011). Phenotypical and functional specialization of FOXP3+ regulatory T cells. Nat. Rev. Immunol. 11, 119–130 10.1038/nri2916 21267013 PMC3289970

[B7] Chan S. S. Schedlich L. J. Twigg S. M. Baxter R. C. (2009). Inhibition of adipocyte differentiation by insulin-like growth factor-binding protein-3. Am. J. Physiol. Endocrinol. Metab. 296, E654–E663 10.1152/ajpendo.90846.2008 19141684

[B8] Chatila T. A. Blaeser F. Ho N. Lederman H. M. Voulgaropoulos C. Helms C. Bowcock A. M. (2000). JM2, encoding a fork head-related protein, is mutated in X-linked autoimmunity-allergic dysregulation syndrome. J. Clin. Invest. 106, R75–R81 10.1172/JCI11679 11120765 PMC387260

[B9] Chen X. Vinkemeier U. Zhao Y. Jeruzalmi D. Darnell J. E. Jr. Kuriyan J. (1998). Crystal structure of a tyrosine phosphorylated STAT-1 dimer bound to DNA. Cell 93, 827–839 10.1016/S0092-8674(00)81443-9 9630226

[B10] Chia D. J. Subbian E. Buck T. M. Hwa V. Rosenfeld R. G. Skach W. R. Shinde U. Rotwein P. (2006). Aberrant folding of a mutant Stat5b causes growth hormone insensitivity and proteasomal dysfunction. J. Biol. Chem. 281, 6552–6558 10.1074/jbc.M510903200 16303763

[B11] Cohen A. C. Nadeau K. C. Tu W. Hwa V. Dionis K. Bezrodnik L. Teper A. Gaillard M. Heinrich J. Krensky A. M. Rosenfeldand R. G. Lewis D. B. (2006). Cutting edge: decreased accumulation and regulatory function of CD4+ CD25(high) T cells in human STAT5b deficiency. J. Immunol. 177, 2770–2774 16920911 10.4049/jimmunol.177.5.2770

[B12] Crispi S. Sanzari E. Monfregola J. De Felice N. Fimiani G. Ambrosio R. D’Urso M. Ursini M. V. (2004). Characterization of the human STAT5A and STAT5B promoters: evidence of a positive and negative mechanism of transcriptional regulation. FEBS Lett. 562, 27–34 10.1016/S0014-5793(04)00166-8 15043997

[B13] Fang P. Kofoed E. M. Little B. M. Wang X. Ross R. J. Frank S. J. Hwa V. Rosenfeld R. G. (2006). A mutant signal transducer and activator of transcription 5b, associated with growth hormone insensitivity and insulin-like growth factor-I deficiency, cannot function as a signal transducer or transcription factor. J. Clin. Endocrinol. Metab. 91, 1526–1534 10.1210/jc.2005-2558 16464942

[B14] Fontenot J. D. Gavin M. A. Rudensky A. Y. (2003). Foxp3 programs the development and function of CD4+ CD25+ regulatory T cells. Nat. Immunol. 4, 330–336 10.1038/ni904 12612578

[B15] Grimley P. M. Dong F. Rui H. (1999). Stat5a and Stat5b: fraternal twins of signal transduction and transcriptional activation. Cytokine Growth Factor Rev. 10, 131–157 10.1016/S1359-6101(99)00011-8 10743504

[B16] Hori S. Nomura T. Sakaguchi S. (2003). Control of regulatory T cell development by the transcription factor Foxp3. Science 299, 1057–1061 10.1126/science.1079490 12522256

[B17] Hwa V. Camacho-Hübner C. Little B. M. David A. Metherell L. A. El-Khatib N. Savageb M. O. Rosenfeld R. G. (2007). Growth hormone insensitivity and severe short stature in siblings: a novel mutation at the exon 13-intron 13 junction of the STAT5b gene. Horm. Res. 68, 218–224 10.1159/000101334 17389811

[B18] Hwa V. Little B. Adiyaman P. Kofoed E. M. Pratt K. L. Ocal G. Berberoglu M. Rosenfeld R. G. (2005). Severe growth hormone insensitivity resulting from total absence of signal transducer and activator of transcription 5b. J. Clin. Endocrinol. Metab. 90, 4260–4266 10.1210/jc.2005-0515 15827093

[B19] Hwa V. Little B. Kofoed E. M. Rosenfeld R. G. (2004). Transcriptional regulation of insulin-like growth factor-I by interferon-gamma requires STAT-5b. J. Biol. Chem. 279, 2728–2736 10.1074/jbc.M310495200 14570891

[B20] Hwa V. Nadeau K. Wit J. M. Rosenfeld R. G. (2011). STAT5b deficiency: lessons from STAT5b gene mutations. Best Pract. Res. Clin. Endocrinol. Metab. 25, 61–75 10.1016/j.beem.2010.09.003 21396575

[B21] John S. Vinkemeier U. Soldaini E. Darnell J. E. Jr. Leonard W. J. (1999). The significance of tetramerization in promoter recruitment by Stat5. Mol. Cell. Biol. 19, 1910–1918 10022878 10.1128/mcb.19.3.1910 PMC83984

[B22] Kawai M. Rosen C. J. (2010). The IGF-I regulatory system and its impact on skeletal and energy homeostasis. J. Cell. Biochem. 111, 14–19 10.1002/jcb.22678 20506515 PMC3276304

[B23] Khattri R. Cox T. Yasayko S. A. Ramsdell F. (2003). An essential role for Scurfin in CD4+ CD25+ T regulatory cells. Nat. Immunol. 4, 337–342 10.1038/ni909 12612581

[B24] Kofoed E. M. Hwa V. Little B. Woods K. A. Buckway C. K. Tsubaki J. Pratt K. L. Bezrodnik L. Jaspe H. Teppe A. Heinrich J. J. Rosenfeld R. G. (2003). Growth hormone insensitivity associated with a STAT5b mutation. N. Engl. J. Med. 349, 1139–1147 10.1056/NEJMoa022926 13679528

[B25] Langlois J. A. Rosen C. J. Visser M. Hannan M. T. Harris T. Wilson P. W. F. Kiel D. P. (1998). Association between insulin-like growth factor I and bone mineral density in older women and men: the Framingham Heart Study. J. Clin. Endocrinol. Metab. 83, 4257–4262 10.1210/jc.83.12.4257 9851760

[B26] Levy D. E. Darnell J. E. Jr. (2002). Stats: transcriptional control and biological impact. Nat. Rev. Mol. Cell. Biol. 3, 651–662 10.1038/nrg893 12209125

[B27] Lin J. X. Leonard W. J. (2000). The role of Stat5a and Stat5b in signaling by IL-2 family cytokines. Oncogene 19, 2566–2576 10.1038/sj.onc.1203924 10851055

[B28] Liu X. Robinson G. W. Wagner K. U. Garrett L. Wynshaw-Boris A. Hennighausen L. (1997). Stat5a is mandatory for adult mammary gland development and lactogenesis. Genes Dev. 11, 179–186 10.1101/gad.11.23.3157 9009201

[B29] Llambi F. Green D. R. (2011). Apoptosis and oncogenesis: give and take in the BCL-2 family. Curr. Opin. Genet. Dev. 21, 12–20 10.1016/j.gde.2010.12.001 21236661 PMC3040981

[B30] Mandal M. Powers S. E. Maienschein-Cline M. Bartom E. T. Hamel K. M. Kee B. L. Dinner A. R. Clark M. R. (2011). Epigenetic repression of the Igk locus by STAT5-mediated recruitment of the histone methyltransferase Ezh2. Nat. Immunol. 12, 1212–1220 10.1038/ni.2136 22037603 PMC3233979

[B31] Nadeau K. Hwa V. Rosenfeld R. G. (2011). STAT5b deficiency: an unsuspected cause of growth failure, immunodeficiency, and severe pulmonary disease. J. Pediatr. 158, 701–708 10.1016/j.jpeds.2010.12.042 21414633

[B32] Pugliese-Pires P. N. Tonelli C. A. Dora J. M. Silva P. C. Czepielewski M. Simoni G. Arnhold I. J. P. Jorge A. A. L. (2010). A novel STAT5B mutation causing GH insensitivity syndrome associated with hyperprolactinemia and immune dysfunction in two male siblings. Eur. J. Endocrinol. 163, 349–355 10.1530/EJE-10-0272 20538865

[B33] Russell S. M. Tayebi N. Nakajima H. Riedy M. C. Roberts J. L. Aman M. J. (1995). Mutation of Jak3 in a patient with SCID: essential role of Jak3 in lymphoid development. Science 270, 797–800 10.1126/science.270.5237.797 7481768

[B34] Sakaguchi S. Sakaguchi N. Asano M. Itoh M. Toda M. (1995). Immunologic self-tolerance maintained by activated T cells expressing IL-2 receptor alpha-chains (CD25). Breakdown of a single mechanism of self-tolerance causes various autoimmune diseases. J. Immunol. 155, 1151–1164 7636184

[B35] Sakaguchi S. Yamaguchi T. Nomura T. Ono M. (2008). Regulatory T cells and immune tolerance. Cell 133, 775–787 10.1016/j.cell.2008.05.009 18510923

[B36] Scaglia P. A. Martínez A. S. Feigerlová E. Bezrodnik L. Gaillard M. I. Di Giovanni D. Ballerini M. G. Jasper H. G. Heinrich J. J. Fang P. Domené H. M. Rosenfeld R. G. Hwa V. (2012). A novel missense mutation in the SH2 domain of the STAT5B gene results in a transcriptionally inactive STAT5b associated with severe IGF-I deficiency, immune dysfunction, and lack of pulmonary disease. J. Clin. Endocrinol. Metab. 97, E830–E839 10.1210/jc.2011-2554 22419735

[B37] Sharfe N. Dadi H. K. Shahar M. Roifman C. M. (1997). Human immune disorder arising from mutation of the alpha chain of the interleukin-2 receptor. Proc. Natl. Acad. Sci. U.S.A. 94, 3168–3171 10.1073/pnas.94.7.3168 9096364 PMC20340

[B38] Soldaini E. John S. Moro S. Bollenbacher J. Schindler U. Leonard W. J. (2000). DNA binding site selection of dimeric and tetrameric Stat5 proteins reveals a large repertoire of divergent tetrameric Stat5a binding sites. Mol. Cell. Biol. 20, 389–401 10.1128/MCB.20.1.389-401.2000 10594041 PMC85094

[B39] Strauss L. Czystowska M. Szajnik M. Mandapathil M. Whiteside T. L. (2009). Differential responses of human regulatory T cells (Treg) and effector T cells to rapamycin. PloS ONE 4, e5994 10.1371/journal.pone.0005994 19543393 PMC2694984

[B40] Tischner D. Woess C. Ottina E. Villunger A. (2010). Bcl-2-regulated cell death signalling in the prevention of autoimmunity. Cell Death Dis. 1, e48 10.1038/cddis.2010.27 21364654 PMC3032315

[B41] Ulukaya E. Acilan C. Yilmaz Y. (2011). Apoptosis: why and how does it occur in biology? Cell Biochem. Funct. 29, 468–480 10.1002/cbf.1774 21773978

[B42] Vidarsdottir S. Walenkamp M. J. Pereira A. M. Karperien M. van Doorn J. van Duyvenvoorde H. A. White S. Breuning M. H. Roelfsema F. Kruithof M. F. van Dissel J. Janssen R. Wit J. M. Romijn J. A. (2006). Clinical and biochemical characteristics of a male patient with a novel homozygous STAT5b mutation. J. Clin. Endocrinol. Metab. 91, 3482–3485 10.1210/jc.2006-0368 16787985

[B43] Wei L. Laurence A. O’Shea J. J. (2008). New insights into the roles of Stat5a/b and Stat3 in T cell development and differentiation. Semin. Cell Dev. Biol. 19, 394–400 10.1016/j.semcdb.2008.07.011 18708155 PMC2657870

[B44] Wildin R. S. Ramsdell F. Peake J. Faravelli F. Casanova J. L. Buist N. Levy-Lahad M. Mazzella E. M. Goulet O. Perroni L. Bricarelli F. D. Byrne G. McEuen M. Proll S. Appleby M. Brunkow M. E. (2001). X-linked neonatal diabetes mellitus, enteropathy and endocrinopathy syndrome is the human equivalent of mouse scurfy. Nat. Genet. 27, 18–20 10.1038/83707 11137992

[B45] Willerford D. M. Chen J. Ferry J. A. Davidson L. Ma A. Alt F. W. (1995). Interleukin-2 receptor alpha chain regulates the size and content of the peripheral lymphoid compartment. Immunity 3, 521–530 10.1016/1074-7613(95)90180-9 7584142

[B46] Xu D. Qu C. K. (2008). Protein tyrosine phosphatases in the JAK/STAT pathway. Front. Biosci. 13:4925–4932 10.2741/2824 18508557 PMC2599796

[B47] Zeiser R. Leveson-Gower D. B. Zambricki E. A. Kambham N. Beilhack A. Loh J. Hou1 J.-Z. Negrin R. S. (2008). Differential impact of mammalian target of rapamycin inhibition on CD4+ CD25+ Foxp3+ regulatory T cells compared with conventional CD4+ T cells. Blood 111, 453–462 10.1182/blood-2007-06-094482 17967941 PMC2200823

[B48] Zhu M. H. Berry J. A. Russell S. M. Leonard W. J. (1998). Delineation of the regions of interleukin-2 (IL-2) receptor beta chain important for association of Jak1 and Jak3. *Jak*1-independent functional recruitment of Jak3 to Il-2Rbeta. J. Biol. Chem. 273, 10719–10725 10.1074/jbc.273.23.14254 9553136

